# A Rare and Intriguing Case of Wilson's Disease Initially Suspected of Systemic Lupus Erythematosus

**DOI:** 10.1002/ccr3.70091

**Published:** 2025-01-07

**Authors:** Mandana Khodashahi, Najmeh Mohajeri, Moeid Reza Alipour, Reza Khademi, Nama Mohamadian Roshan, Behzad Aminzadeh, Muhammed Joghatayi

**Affiliations:** ^1^ Rheumatic Diseases Research Center, Ghaem Hospital, Taghi Abad Mashhad University of Medical Sciences Mashhad Iran; ^2^ Department of Pathology, Ghaem Hospital, Taghi Abad Mashhad University of Medical Sciences Mashhad Iran; ^3^ Department of Radiology, Faculty of Medicine, Vakil Abad Mashhad University of Medical Sciences Mashhad Iran

**Keywords:** hepatomegaly, liver injury, pancytopenia, systemic lupus erythematosus, Wilson's disease

## Abstract

When systematic lupus erythematosus‐like lab results (e.g., positive anti‐double‐stranded DNA antibody, low complement component 3) are inconsistent with physical findings, such as the absence of arthritis or nephritis, clinicians should consider diagnoses such as Wilson's disease, especially in the presence of abnormal liver function and elevated international normalized ratio (INR).

## Graphical abstract

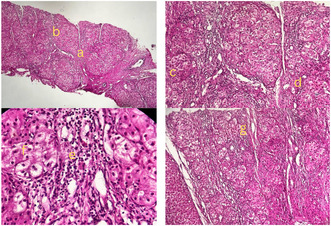

## 1 Introduction

Wilson's disease (WD) is a rare autosomal‐recessive disorder characterized by defective copper metabolism. Copper accumulation starts at birth, and patients usually become symptomatic in adolescence or early adulthood. Symptoms arise due to copper buildup in various organs, particularly the liver and brain [1].

Systemic lupus erythematosus (SLE) is a multisystem autoimmune disease that can affect any organ. It is also known as the “*Great Imitator*” [2] due to its vast variability in presentations, signs, and symptoms. Despite significant research, the diagnosis and management of SLE remain complex and challenging. It can manifest in a wide array of clinical forms, ranging from mild fatigue to severe complications such as acute kidney injury, psychosis, and seizures [3].

Our patient was referred to the rheumatology clinic because of positive paraclinical tests for SLE, pancytopenia, elevated liver enzymes, and fatigue, which can all be seen in possible cases of SLE.

This case report underscores the diagnostic complexities when distinguishing WD from SLE, particularly in patients presenting with hepatic and hematologic abnormalities commonly associated with both conditions.

## 2 Case History

A 19‐year‐old girl with no significant previous medical history except for a low mood and depression, with a suicide attempt 6 months prior, medication use, or illicit drug use, was referred to an internist due to a 2‐year history of fatigue, asthenia, easy bruising, and occasional epistaxis. These symptoms had been gradually worsening over the past 6 months. She had no significant family history among her first‐ and second‐degree relatives, and her parents were non‐consanguineous. Routine tests revealed pancytopenia (white blood cell (WBC) = 3.3 × 10^9/L, red blood cell (RBC) = 3.8 × 10^12/L, hemoglobine (Hb) = 10.9 g/dL, and platelets (PLT) = 59 × 10^9/L), normal serum creatine level of 0.9 mg/dL (normal range = 0.6–1.3), elevated liver enzymes (aspartate aminotransferase (AST) = 214 U/L [normal range: up to 38], alanine aminotransferase (ALT) = 196 U/L [normal range: up to 31], alkaline phosphatase (ALP) = 442 U/L [normal range: up to 480]), total bilirubin of 3 mg/dL, direct bilirubin of 1 mg/dL, and lactate dehydrogenase (LDH) of 418 U/L [normal range: up to 480], along with disturbed coagulation tests (partial thromboplastin time (PTT) = 49.4 s [normal range: 25–35], INR 1.52, prothrombin time (PT) = 22.4 s). She and her parents had no history of jaundice.

The conjugated hyperbilirubinemia increased AST and ALT, and normal ALP suggested hepatocellular damage. Considering her age, the internist ordered a viral hepatitis panel (antibody to hepatitis A virus (Anti‐HAV), hepatitis B core antibody (HBc Ab), hepatitis C virus antibody (HCV Ab), and hepatitis B surface antigen (HBs Ag)) and markers for rheumatologic diseases and autoimmune hepatitis. The viral panel results were negative, but fluorescent antinuclear antibody (FANA), Anti‐Ro, and anti‐cyclic citrullinated peptide antibodies (Anti‐CCP) were positive, while anti‐mitochondrial antibodies (AMA) was negative. She had an rheumatoid factor (RF) (55 IU/mL [normal range: up to 20]) and erythrocyte sedimentation rate (ESR) (20 mm/h), with C‐reactive protein (CRP) within the normal range (0.2 mg/dL [normal range: up to 6]). Urinalysis showed no hematuria or proteinuria. The internist referred the patient to a rheumatology clinic for further investigation.

Except for fatigue, her physical examination and history‐taking revealed no significant findings. There was no history of arthritis, arthralgia, seizures, malar rash, skin lesions, or morning stiffness. The physical examination was normal; no findings suggested SLE or rheumatoid arthritis (RA). The pull test was negative, and no mouth ulcers were found.

**FIGURE 1:**
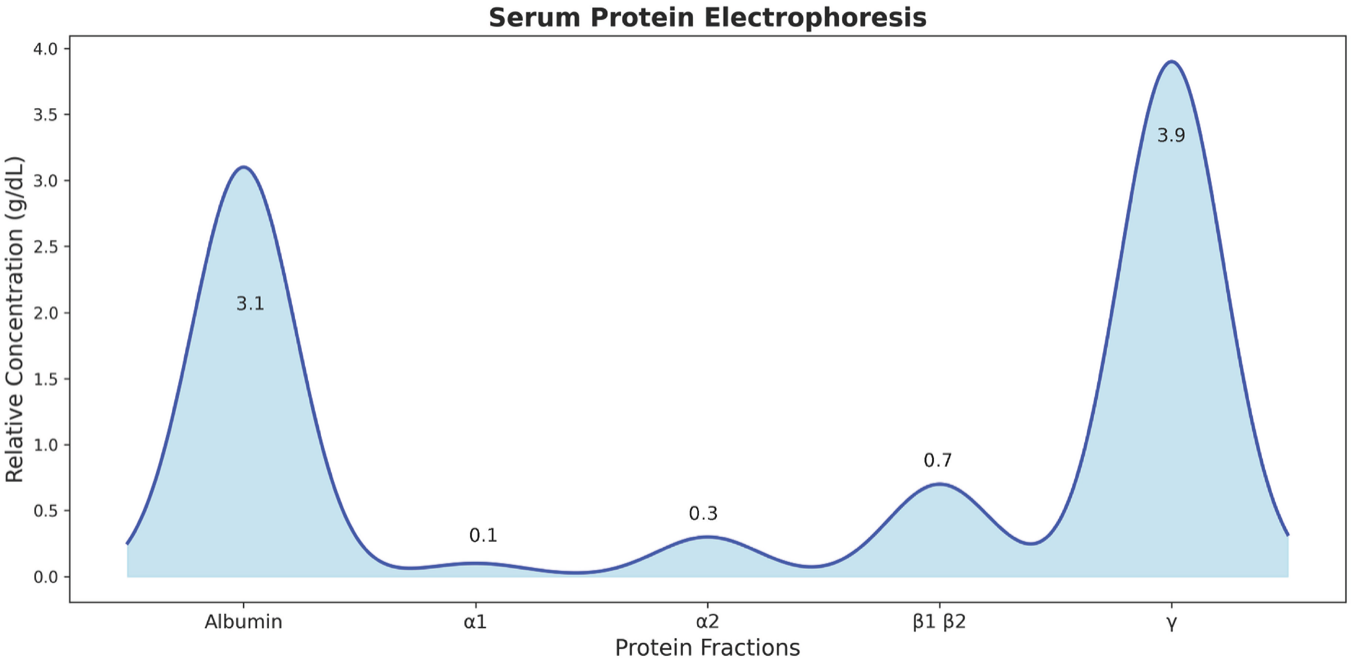
Serum protein electrophoresis shows an increase in γ band and a decrease in albumin level.

Considering her coagulation disturbances, positive ANA profile, high liver enzyme levels, and history of easy bruising and epistaxis, the rheumatologist requested another complete blood count (CBC) and ANA profile, serum levels of C3 and C4, anti‐smooth muscle antibodies (ASMA), anti‐liver kidney microsomal antibodies (anti‐LKM1), perinuclear antinuclear neutrophil antibodies (P‐ANNA), liver and spleen sonography, and a liver coagulation panel (factors VIII, IX, X, and XI activity levels). The results showed a negative anti‐dsDNA and Anti‐Ro, low C3 serum level, normal C4 serum level, and confirmed pancytopenia (WBC = 1.7 × 10^9/L, RBC = 3.7 × 10^12/L, Hb = 10.5 g/dL, PLT = 59 × 10^9/L) and elevated liver enzymes (AST = 218 U/L, ALT = 180 U/L, ALP = 201 U/L). While Anti‐CCP, RF, ASMA, anti‐LKM1, and P‐ANNA were negative. Abdominal sonography showed hepatomegaly with coarse and heterogeneous echogenicity and splenomegaly with normal echogenicity. The activity levels of factors IX, X, and X1 were low, while factor VIII had a high activity level with no response to the mixing test. Serum protein electrophoresis showed low albumin and increased immunoglobulin G (IgG) levels (Figure 1).

Due to her physical examinations and lab data incompatibility, the rheumatologist admitted the patient to the rheumatology unit for further investigations.

## 3 Methods

**FIGURE 2:**
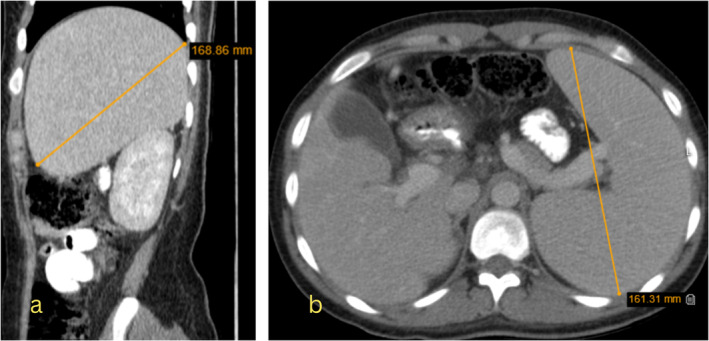
Non‐contrast CT scan with mildly heterogenous liver, which appears normal on the post‐contrast image with enlargement of the spleen. (a) sagittal view, (b) axial view.

She received prednisolone 1 mg/kg/day and intravenous immunoglobulin (IVIG) for 6 days. However, her pancytopenia and high liver enzyme levels did not improve. To rule out cirrhosis, an abdominal CT scan with and without injection and color‐doppler ultrasound of the portal vein was performed. An endoscopy was also performed to check for esophageal varices; no varices were found. The color‐doppler ultrasound showed no thrombosis, normal blood flow, and portal vein diameter. The abdominal CT scan showed hepatomegaly with heterogeneous parenchymal density, an enlarged spleen with normal density, and several lymphadenopathies in para‐aortic and retrocaval regions, suggesting lymphoma (Figure 2).

However, the patient had no history of B symptoms (unintended weight loss, low appetite, or night sweats), and peripheral blood smear (PBS) showed no abnormal white blood cells. Bone marrow aspiration showed normal bone marrow activity, suggesting peripheral pancytopenia.

On the seventh day of her admission, she became gradually agitated and aggressive, had a generalized tonic–clonic seizure, experienced a decrease in consciousness level, and went into a coma (GCS = 3), leading to immediate Intensive Care Unit (ICU) admission. The vital signs were normal. Her pulse rate was 80/min. Her respiratory rate was 22/min. Her blood pressure was 126/71 mmHg, and her body temperature was 36.6°C. Her oxygen saturation was 98% on room air. Lab data showed within normal range serum electrolytes (Na = 138 [normal range: 135–145 mEq/L] K = 3.7 [normal range: 3.5–5.3 mEq/L]) and a mild respiratory alkalosis (pH = 7.49, pCO_2_ = 29 mmHg and HCO3 = 23.3). A brain CT scan showed effacement and slit in both lateral ventricles with brain edema and no mass lesion, prompting an MRI due to her acute condition.

**FIGURE 3:**
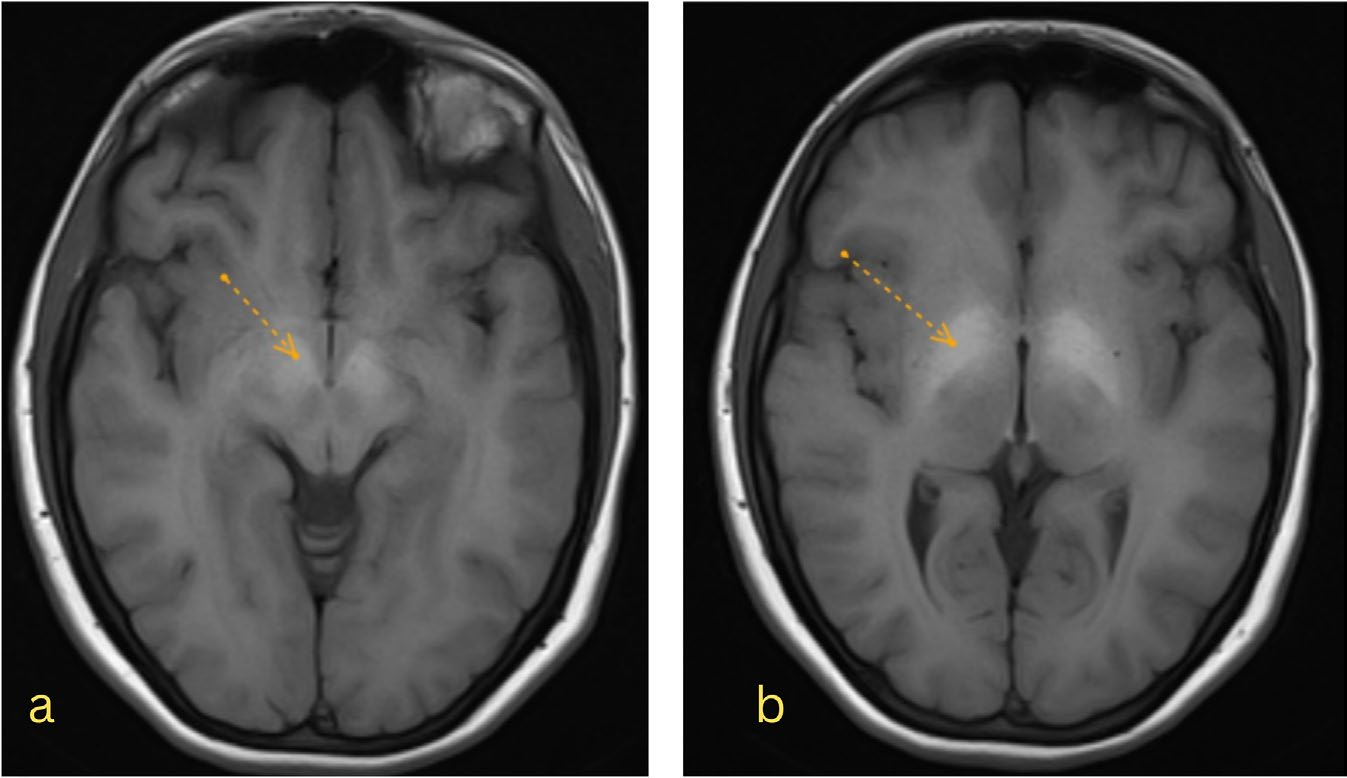
Symmetrical high T1 signal is seen within the bilateral globus pallidi with a normal appearance on T2W images. (a) hyperintensity on bilateral globi pallidi on T1W images. (b) hyperintensity in corticospinal (pyramidal) tract.

A neurology consult requested a brain magnetic resonance imaging (MRI) with and without injection and MRV, revealing hypersignal intensity in both globus pallidi on T1‐weighted images without enhancement and restriction, compatible with toxic or metabolic encephalopathy (Figure 3). No abnormality was seen after the gadolinium (GD) injection. Magnetic resonance venography (MRV) showed no thrombosis and was reported as normal.

Ophthalmologic examination was negative for Kayser‐Fleischer (KF) rings or sunflower cataracts.

**FIGURE 4:**
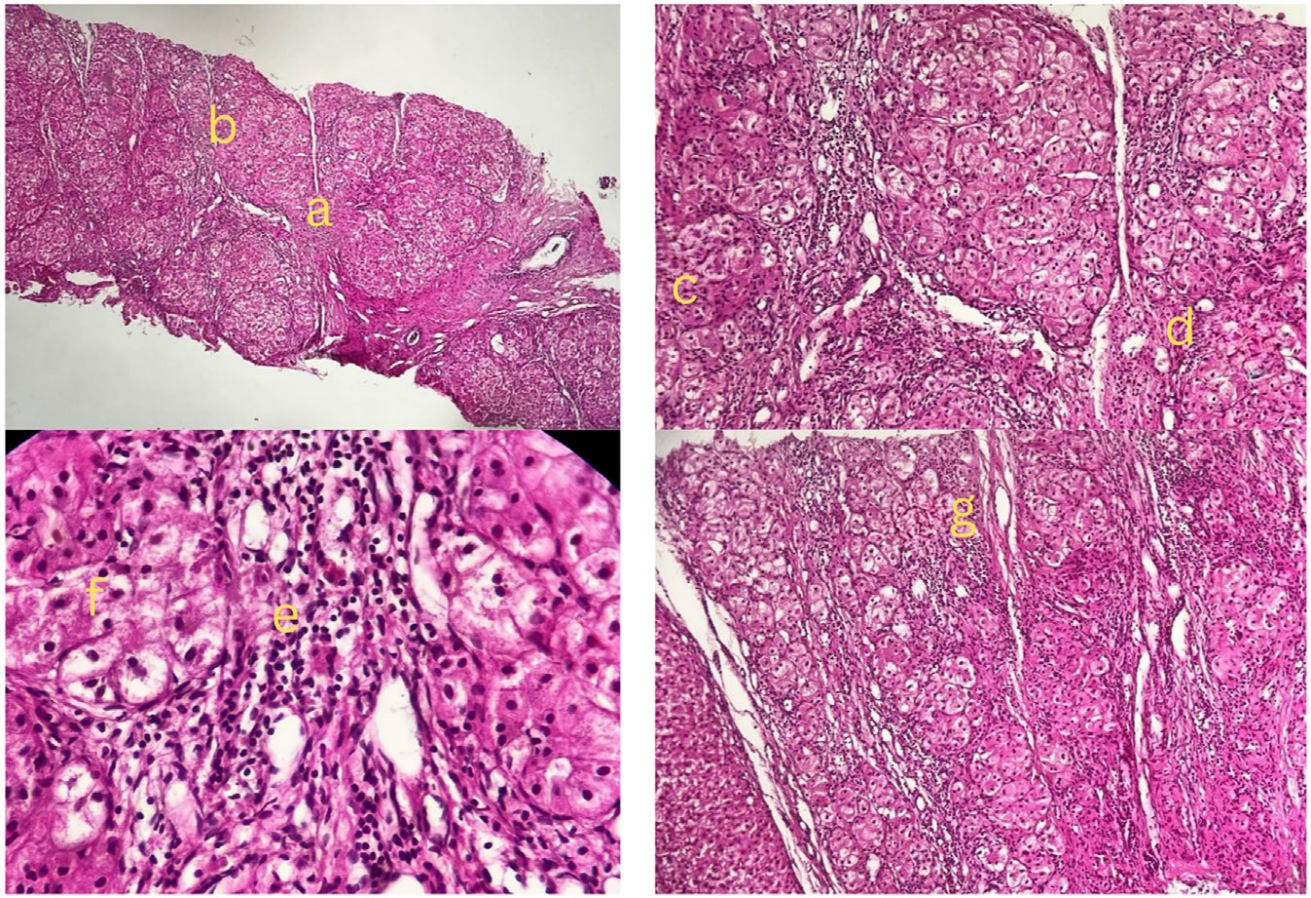
The patient's liver biopsy showed nodular liver and fibrosis with portal and lobular inflammation. (a) Inter‐nodular fibrosis, (b) nodular pattern, (c) hepatocytic Rosette, (d) hepatocyte ballooning, (e) lympho‐plasma cell infiltration, (f) hepatocyte damage, (g) mild cholestasis.

After correcting the INR with vitamin K and receiving multiple fresh frozen plasma (FFP) and platelet transfusions, a lumbar puncture to rule out septic meningoencephalitis, including Tuberculosis (TB) and Herpes simplex virus polymerase chain reaction (HSV PCR), was performed and came back negative. Given the paraclinical results indicating hepatic encephalopathy, she was listed for a liver transplant and was treated with mannitol and lactulose in the ICU. She fully recovered and became conscious and oriented after 24 h. A liver biopsy revealed nodular liver and fibrosis with portal and lobular inflammation, suggesting metabolic disorders such as wilson's disease or autoimmune hepatitis (Figure 4).

Further copper concentration measurement in the liver biopsy showed a high level of copper (260 μg/g dry weight [normal range: up to 50 μg/g]). Serum ceruloplasmin was low (157 μg/dL [normal range: 204–407 μg/dL]), and 24‐h urine copper excretion was high (168 μg/24 h [normal range: 15–70 μg/24 h]), confirming wilson's disease. The summary of laboratory tests, imaging studies, and their findings, used to evaluate the patient's condition are presented in Table 1.

**TABLE 1:** Diagnostic workup timeline.

Date	Tests performed	Findings	Normal range
Initial lab tests ordered by internist	CBC	WBC = 3.3 × 10^9/L RBC = 3.8 × 10^12/L PLT = 59 × 10^9/L	4.5–11.0 × 10^9/L 4.2–5.4 × 10^12/L 150–400 × 10^9/L
	Liver enzymes	AST = 214 U/L ALT = 196 U/L ALP = 442 U/L	Up to 38 Up to 31 Up to 480
	Coagulation tests	PTT 49.4 s INR 1.52 PT = 22.4 s	25–35 s—11–13.5 s
Follow‐up tests ordered by internist	FANA	Positive	Qualitative
	Anti‐CCP	Positive	Qualitative
	RF	55 IU/mL (elevated)	Up to 20
	Anti‐dsDNA	Negative	Qualitative
	Anti‐Ro	Positive	Qualitative
	Urinalysis	No hematuria or proteinuria	—
	Viral hepatitis panel (Anti‐HAV, HBc Ab, HCV Ab, HBs Ag)	All negative	—
	Autoimmune hepatitis biomarkers	All negative	Qualitative
Tests ordered by rheumatologis	CBC	WBC = 1.7 × 10^9/L RBC = 3.7 × 10^12/L PLT = 59 × 10^9	4.5–11.0 × 10^9/L 4.2–5.4 × 10^12/L 150–400 × 10^9/L
	Liver enzymes	AST = 214 U/L ALT = 196 U/L ALP = 442 U/L	Up to 38 Up to 31 Up to 480
	VIII, IX, X, and XI activity levels	VIII (elevated) IX, X, and XI (decreased)	—
	ANA profile	All negative	—
	Abdominal sonography	Hepatomegaly Splenomegaly	—
Hospital admission tests	MRI	Hypersignal intensity in globi pallidi	—
	Endoscopy	No varices	—
	Color‐doppler ultrasound of portal vein	No thrombosis Normal flow	—
	Abodmianl CT scan	Hepatomegaly Splenomegaly Para‐aortic lymphadenopathy	—
	Bone marrow aspiration	Normal bone marrow activity	—
Copper studies and liver biopsy	Urinary copper	168 μg/24 h (elevated)	15–70 μg/24 h
	Serum ceruloplasmin	57 μg/dL (low)	204–407 μg/dL
	Liver biopsy	260 μg/g (elevated)	Up to 50 μg/g dry weight
Follow‐up	CBC	WBC = 6.28 × 10^9/L RBC = 3.47 × 10^12/L PLT = 70 × 10^9/L	4.5–11.0 × 10^9/L 4.2–5.4 × 10^12/L 150–400 × 10^9/L
	Liver enzymes	AST = 33 U/L ALT = 110 U/L (elevated) ALP = 498 U/L	Up to 38 Up to 31 Up to 480

## 4 Conclusion and Results

Following the confirmation of wilson's disease, treatment was initiated with zinc sulfate (25 mg three times daily) and trientine (1250 mg/day) to reduce copper accumulation and promote its excretion. The patient's pancytopenia and coagulation abnormalities showed progressive improvement, indicating a favorable response to treatment (WBC = 6.28 × 10^9/L, RBC = 3.47 × 10^12/L, PLT = 70 × 10^9/L, PT = 34 s, PTT = 35, INR = 1.11). Her liver enzymes began to normalize (AST = 33 U/L, ALT = 110 U/L, ALP = 498 U/L). She was discharged 5 days later with a good general well‐being. During her regular examinations, she showed significant improvement.

## 5 Discussion

This case report describes a 19‐year‐old female initially suspected to have SLE due to her symptoms of fatigue, pancytopenia, elevated liver enzymes, and positive autoimmune markers. However, further investigations revealed WD as the correct diagnosis, highlighted by abnormal liver biopsy findings, high hepatic copper content, low serum ceruloplasmin, and elevated 24‐h urine copper excretion.

WD primarily targets the liver and brain, similar to SLE, which can present with neurological and hepatic manifestations [1, 3].

While the involvement of SLE in the onset of asymptomatic liver disease remains a topic of debate, many experts acknowledge that SLE frequently results in subclinical liver dysfunction, referred to as lupus hepatitis. This condition is a nonspecific reactive liver disease, primarily driven by complement deposition and vasculitis‐induced damage to the liver. Research indicates that hepatomegaly is present in about 20%–40% of SLE patients and is often associated with autoimmune hepatitis, lupus hepatitis, or drug‐induced liver injury. However, hepatomegaly is relatively uncommon in SLE and typically signals the coexistence of autoimmune or drug‐induced hepatitis [4].

In this case, specific clinical and laboratory findings did not match with SLE or autoimmune hepatitis. Although PTT can be abnormal in SLE, the presence of high INR, along with a coarsely enlarged liver [5, 6], led the rheumatologist to lean more toward a liver injury rather than SLE: the negative AMA, ASMA, and P‐ANNA. Anti‐LKM1, coupled with no response to corticosteroids, significantly lowered the probability of autoimmune hepatitis [7].

This case presented several diagnostic pitfalls. Positive ANA and RF can also be seen in WD, but anti‐Ro antibodies and low C3 levels are not typically reported in WD [8] and mislead the diagnosis. The absence of KF rings in the eyes, a hallmark of WD [1], was another diagnostic challenge. Additionally, symptoms like pancytopenia, fatigue, and a history of depression are common in both WD and SLE, complicating the diagnosis further [1, 9]. Moreover, the patient's age (19 years) posed a diagnostic pitfall since the onset of both diseases typically occurs in early adulthood.

Notably, WD has been associated with a range of immunological abnormalities, including positive ANA, positive anti‐neutrophil cytoplasmic antibody (ANCA), and anti‐CCP. Such markers, typically linked to autoimmune diseases such as SLE, autoimmune hepatitis, and multiple sclerosis, can complicate diagnosis by mimicking or coexisting with autoimmune conditions [10].

For example, anti‐Ro antibodies, primarily associated with Sjögren's syndrome and occasionally found in SLE, can appear at low titers in about 15% of healthy individuals. Though less common, false positives remain a possibility [11].

This immunological overlap becomes even more complex in WD patients treated with D‐penicillamine, a drug linked to drug‐induced lupus [12], which increases the risk of misdiagnosis. Therefore, the presence of these autoantibodies in patients with WD, especially those receiving D‐penicillamine, requires careful interpretation, such as assessing coexisting antibodies like Anti‐histone antibodies (AHA), to differentiate between underlying autoimmune processes and drug effects rather than assuming a definitive autoimmune disease.

Low complement levels, particularly C3, in SLE, are primarily due to immune complex formation that activates and consumes complement proteins, and they are mainly suggestive of high disease activity [13]. However, complement levels can also be reduced due to liver dysfunction, as the liver synthesizes these proteins, a key pathology in WD [14]. In the absence of SLE symptoms and signs of high activity, such as lupus nephritis or arthritis, other mechanisms, including liver dysfunction, should be considered. It is crucial not to attribute low complement levels solely to autoimmune processes in the absence of signs and symptoms of SLE.

Several case reports have documented the overlap of SLE and WD, complicating the diagnosis and treatment strategies. For instance, Hadef et al. reported a case of a 12‐year‐old boy with concurrent WD and SLE, where both conditions were diagnosed simultaneously. The patient initially presented with hemolytic anemia and impaired liver function, leading to the suspicion of WD. Further investigations confirmed both WD and SLE, and the patient responded to treatment with corticosteroids and chelation therapy [15].

Zhang et al. described a 35‐year‐old woman with SLE who was later found to have WD during her routine follow‐up due to unexplained liver fibrosis. Genetic testing confirmed WD, and the patient was treated with zinc sulfate and medications for SLE. This case underscores the importance of considering WD in patients with unexplained hepatic involvement in SLE [16].

Yang et al. presented a case of a 9‐year‐old girl diagnosed with both SLE and WD. Despite effective control of her SLE symptoms, her liver function did not improve, leading to the suspicion of WD. Genetic testing confirmed the diagnosis, and the patient underwent liver transplantation. Unfortunately, she passed away shortly after the surgery, highlighting the severe implications of delayed diagnosis and treatment [17].

However, the patient did not respond to SLE treatment in this case. In contrast, WD treatment with zinc sulfate and trientine significantly improved pancytopenia, elevated liver enzymes, and impaired INR. This response indicates that the case was not an overlap of SLE and WD but rather a misdiagnosis of SLE in the presence of WD.

This case highlights one key point: positive ANA profile results alone are insufficient to diagnose SLE. Physicians must consider other laboratory data, such as impaired INR and hepatomegaly, which are uncommon in SLE.

The absence of typical physical examination findings should be strongly considered, as emphasized in the 2019 EULAR/ACR (2019 European League Against Rheumatism/American College of Rheumatology) criteria [18]. Physicians should avoid unnecessary lab tests that can mislead the diagnostic approach and increase the burden on the healthcare system [19]. The case emphasizes the importance of comprehensive clinical evaluation and the interpretation of laboratory tests in the context of the patient's overall condition [20]. Highlighting the famous quote in medicine: “Treat the patient, not the disease” [21].

## 6 Conclusion

This case report underscores the diagnostic challenges in distinguishing between SLE and WD. It emphasizes the importance of thorough investigation and consideration of WD in patients with hepatic and hematologic abnormalities unresponsive to conventional treatments for autoimmune diseases. Early diagnosis and appropriate treatment of WD are essential to improve patient outcomes and prevent irreversible organ damage.

## Author Contributions

**Mandana Khodashahi:** conceptualization, data curation, investigation, project administration, supervision, validation, writing – review and editing. **Najmeh Mohajeri:** data curation, investigation, validation, writing – review and editing. **Moeid Reza Alipour:** data curation, investigation, writing – review and editing. **Reza Khademi:** data curation, investigation, writing – review and editing. **Nama Mohamadian Roshan:** investigation, methodology, validation, writing – original draft. **Behzad Aminzadeh:** investigation, methodology, validation, writing – original draft. **Muhammed Joghatayi:** conceptualization, data curation, investigation, project administration, validation, visualization, writing – original draft, writing – review and editing.

## Ethics Statement

This case report was conducted in accordance with the principles of the Declaration of Helsinki. All efforts were made to maintain the patient's privacy and confidentiality.

## Consent

Written informed consent was obtained from the patient for the publication of this case report, including clinical details and images.

## Conflicts of Interest

The authors declare no conflicts of interest.

## Data Availability

Data sharing not applicable to this article as no datasets were generated or analyzed during the current study.
